# Building better polymerases: Engineering the replication of expanded genetic alphabets

**DOI:** 10.1074/jbc.REV120.013745

**Published:** 2020-10-01

**Authors:** Zahra Ouaray, Steven A. Benner, Millie M. Georgiadis, Nigel G. J. Richards

**Affiliations:** 1 School of Chemistry, Cardiff University, Park Place, Cardiff, United Kingdom; 2 Foundation for Applied Molecular Evolution, Alachua, Florida, USA; 3 Department of Biochemistry and Molecular Biology, Indiana University School of Medicine, Indianapolis, Indiana, USA

**Keywords:** biotechnology, computer modeling, directed evolution, DNA polymerase, enzyme mechanism, enzyme structure, molecular dynamics, nucleoside/nucleotide analogue, protein-DNA interaction, synthetic biology, X-ray crystallography, expanded genetic alphabets

## Abstract

DNA polymerases are today used throughout scientific research, biotechnology, and medicine, in part for their ability to interact with unnatural forms of DNA created by synthetic biologists. Here especially, natural DNA polymerases often do not have the “performance specifications” needed for transformative technologies. This creates a need for science-guided rational (or semi-rational) engineering to identify variants that replicate unnatural base pairs (UBPs), unnatural backbones, tags, or other evolutionarily novel features of unnatural DNA. In this review, we provide a brief overview of the chemistry and properties of replicative DNA polymerases and their evolved variants, focusing on the Klenow fragment of *Taq* DNA polymerase (Klentaq). We describe comparative structural, enzymatic, and molecular dynamics studies of WT and Klentaq variants, complexed with natural or noncanonical substrates. Combining these methods provides insight into how specific amino acid substitutions distant from the active site in a Klentaq DNA polymerase variant (ZP Klentaq) contribute to its ability to replicate UBPs with improved efficiency compared with Klentaq. This approach can therefore serve to guide any future rational engineering of replicative DNA polymerases.

DNA polymerases (1), RNA polymerases (2), and reverse transcriptases (3) are enzymes central to terran biology and permit life to propagate and exploit its genetic inheritance. DNA polymerases are also workhorses in biotechnology (4, 5), where they often must manage DNA molecules having unusual structural features. For example, some WT bacterial polymerases can replicate DNA-containing UBPs that interact by steric complementarity without any interbase hydrogen bonding (6) (Fig. 1). This finding has been exploited to obtain engineered strains of *Escherichia coli* that can express proteins built from noncanonical amino acids with improved efficiency (15, 16). This capability was a driver of the recent $2.5 billion acquisition of Synthorx by Sanofi-Aventis.

**Figure 1.**
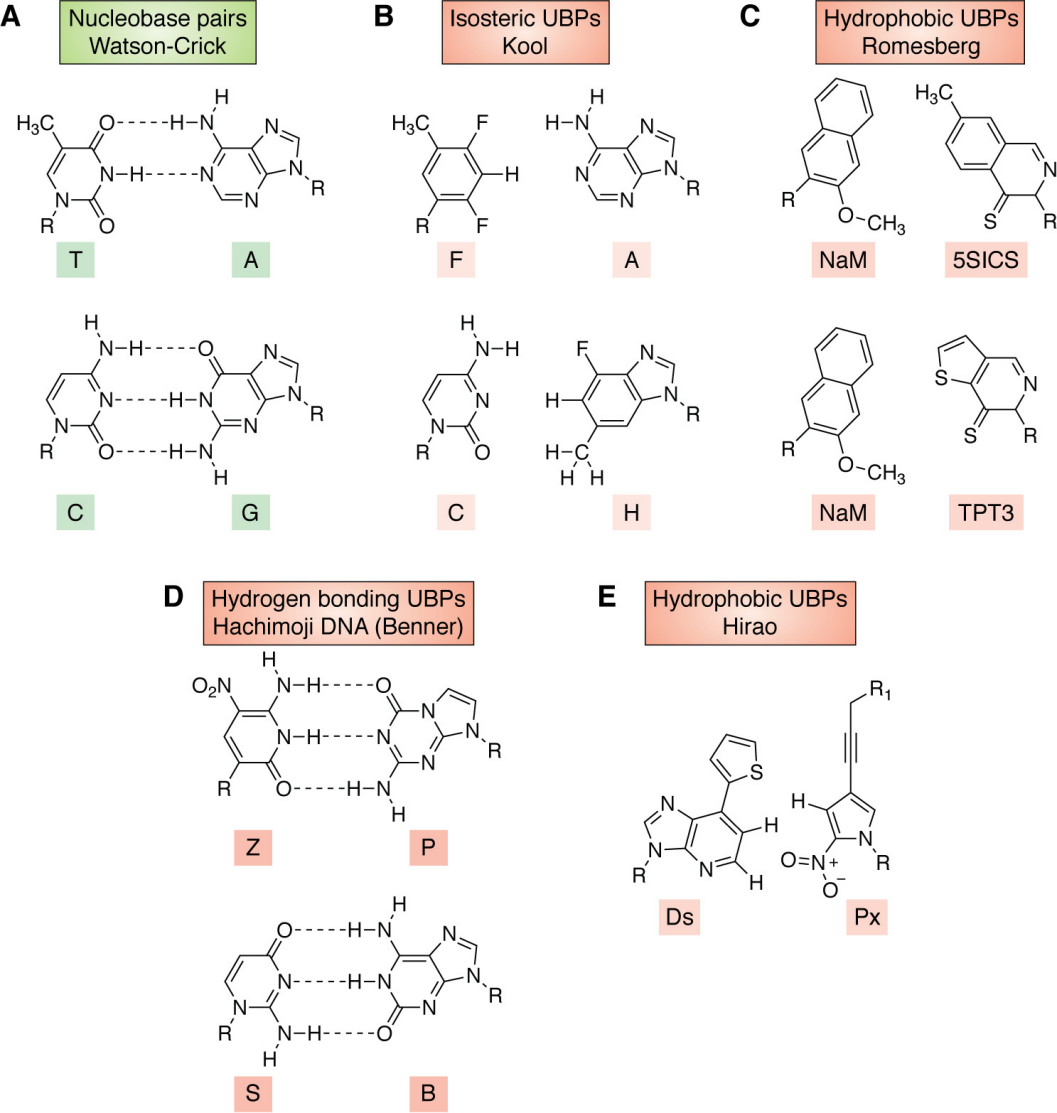
**Chemical structures of nucleobase pairs.** R indicates the point of covalent attachment to either deoxyribose or ribose in DNA or RNA, respectively. *A*, Watson–Crick nucleobase pairs (7). *B*, isosteric UBPs developed by Kool and co-workers (8). *C*, hydrophobic UBPs developed by Romesberg and co-workers (9, 10). *D*, hydrogen-bonding UBPs used in hachimoji DNA developed by Benner and co-workers (11, 12). *E*, hydrophobic UBPs developed by Hirao and co-workers (13, 14), where R_1_ can be a variety of substituents.

DNA polymerase variants are also playing key roles at other scientific frontiers. For example, NextGen sequencing (17) has transformed medicine (18), but only because it was enabled by the use of DNA polymerase variants capable of replicating “Watson–Crick” (WC) deoxynucleoside triphosphates (7) “tagged” with fluorescent markers and carrying blocking groups appended to their 3′-positions (19).

## Expanded genetic alphabets and engineered DNA polymerases

These prior studies, however, only “scratch the surface” of the possible modified nucleotides that have scientific, biotechnological, and medical value. For example, DNA can have many more independently replicable groups than A, T, G, or C (Fig. 1). Scientifically, these groups can be varied to learn whether WC DNA represents the only molecular solution for the long-term storage and replication of biological information (11, 20–22). Alexander Rich was likely the first to recognize the importance of this question, suggesting in 1962 that the isocytidine:isoguanine (S:B) pair (Fig. 1) might be used as an additional information storing unit in DNA (23).

Further, many have asked whether the sugar and phosphate components of the DNA backbone might be modified. Synthetic biologists have therefore synthesized numerous such modified DNA molecules to delineate molecular changes that can (and cannot) be tolerated by duplex DNA (24–26).

The extent to which the core concept of WC pairing can be changed has also been explored by synthetic biologists. For example, the laboratories of Eric Kool (8), Floyd Romesberg (9, 10), and Ichiro Hirao (13, 14) have attempted to dispense with interbase hydrogen bonding (Fig. 1).

Developing a different approach, Steven Benner and co-workers (11, 12) synthesized UBPs that exploit orthogonal patterns of hydrogen bonding. This led to multiple discoveries about the molecular changes that can (and cannot) be tolerated in replicating units, especially how the fidelity of replication can be influenced by protonation, deprotonation, and multiple tautomeric forms of the nucleobases (27). These efforts led to the preparation of “hachimoji” DNA (28), an artificially expanded genetic system (AEGIS) in which A, G, T, and C are augmented by two unnatural pyrimidine analogs, pseudocytidine (or isocytidine) (S) and 6-amino-3-(2′-deoxyribo-furanosyl)-5-nitro-1H-pyridin-2-one (Z), and their size and hydrogen bond complementary partners, the purine analogs, isoguanine (B), and 2-amino-8-(1-β-d-2′-deoxyribofuranosyl)imidazo[1,2-a]-1,3,5-triazin-[8H]-4-one- (P) (27, 28) (Fig. 1). The additional P:Z and B:S base pairs form three interbase hydrogen bonds, as does the natural G:C. Work in this area has also been driven by commercial factors; the use of AEGIS UBPs as components of diagnostic tests has generated lifetime sales in excess of $1 billion (29).

Future disruptive technologies exploiting these and other expanded genetic alphabets will almost certainly require engineered DNA polymerase variants having expanded ranges of biophysical and catalytic properties. Unfortunately, and despite extensive research into the structure and mechanism of DNA polymerases from a wide range of organisms (30–36), rationally predicting how specific amino acid replacements in these enzymes will impact the incorporation of any particular kind of UBP into duplex DNA remains almost impossible. This reflects a lack of fundamental understanding about how multiple factors act synergistically to control UBP incorporation efficiency and fidelity (30, 37, 38). These factors include nucleobase pair complementarity, nucleobase tautomerism, variations in hy-drogen bond free energies, and the conformational dynamics of the polymerase itself as incorporation proceeds through the catalytic cycle. Directed evolution methods (39) are therefore viewed as the best approach to identifying DNA polymerase variants that can incorporate UBPs with high efficiency and fidelity (40–42).

These facts make it timely to review how X-ray crystallography, computational modeling, and directed evolution strategies can be combined to obtain DNA polymerases capable of replicating artificial DNA containing UBPs. Given the recent publication of a comprehensive discussion about the replication of UBP-containing DNA by unengineered WT polymerases (43), we draw on work from our laboratories, the only studies (to our knowledge) that employ a combined experimental and computational approach.

Generally speaking, enzymes that replicate DNA must manage three contradictory demands as they catalyze their critical reactions. First, this class of polymerases must be highly specific, making not much more than one mistake every billion turnovers (44). Mistakes mean somatic mutations potentially leading to cancer, germ line mutations that create inherited genetic disease, and, ultimately, an error catastrophe that leads to the death of the organism. Typical replicative polymerases achieve specificity at both the elongation step and a proofreading step, the latter in the form of 3′–5′ exonuclease domains (see below). Other replicative DNA polymerases, such as reverse transcriptases (45), lack proofreading and are therefore more error-prone. DNA polymerases that function primarily in DNA repair, including those important for genome stability and trans-lesion synthesis, also often have lower fidelity (46, 47).

Second, DNA polymerases must accept four different substrates: (i) template dG and dCTP, (ii) template dC and dGTP, (iii) template dA and dTTP, and (iv) template dT and dATP. Most other enzymes accept only one.

Finally, DNA polymerases must work rapidly. An *E. coli* cell replicates its entire genome by copying 4000 nucleotides each second. Even though many replication forks are used, this rate is as fast as that of many enzymes using only one substrate in pathways able to tolerate mistakes.

Managing these demands is especially difficult because the four substrates have few molecular features in common to present to the polymerase (Fig. 1). For example, the four nucleobases present different functionalities to the major groove, a methyl group for T, a hydrogen for C, two hydrogen bond acceptors for G, and one donor and one acceptor for A.

One feature that all four substrates have in common is their relative size, leading to the rule that large purines must pair with small pyrimidines. Further, the functional groups located on the sides of all four bases in the minor groove of the DNA duplex share a common feature: electron density presented by the exocyclic C=O groups of T and C and the N-3 nitrogen in the purine rings of A and G. Thus, the “minor groove scanning hypothesis” proposes that polymerases donate hydrogen bonds to this electron density in the template, primer, and incoming triphosphate as a way of enforcing Watson–Crick geometry, and hence fidelity, on base pair recognition (48).

These hydrogen bond contacts and size constraints are well-represented in over two dozen crystal structures solved to date (32, 43). The electronic constraint in the minor groove also appears to be important for nonstandard base pairing, even pairing that, as in the Romesberg pair (Fig. 1), involves no interbase hydrogen bonding (22). Moreover, the easiest AEGIS UBP to incorporate into DNA by polymerase-catalyzed reactions has been Z:P (Fig. 1), which is the only pair of those with shuffled hydrogen-bonding patterns for which both components present electron density to the minor groove (49).

**Figure 2.**
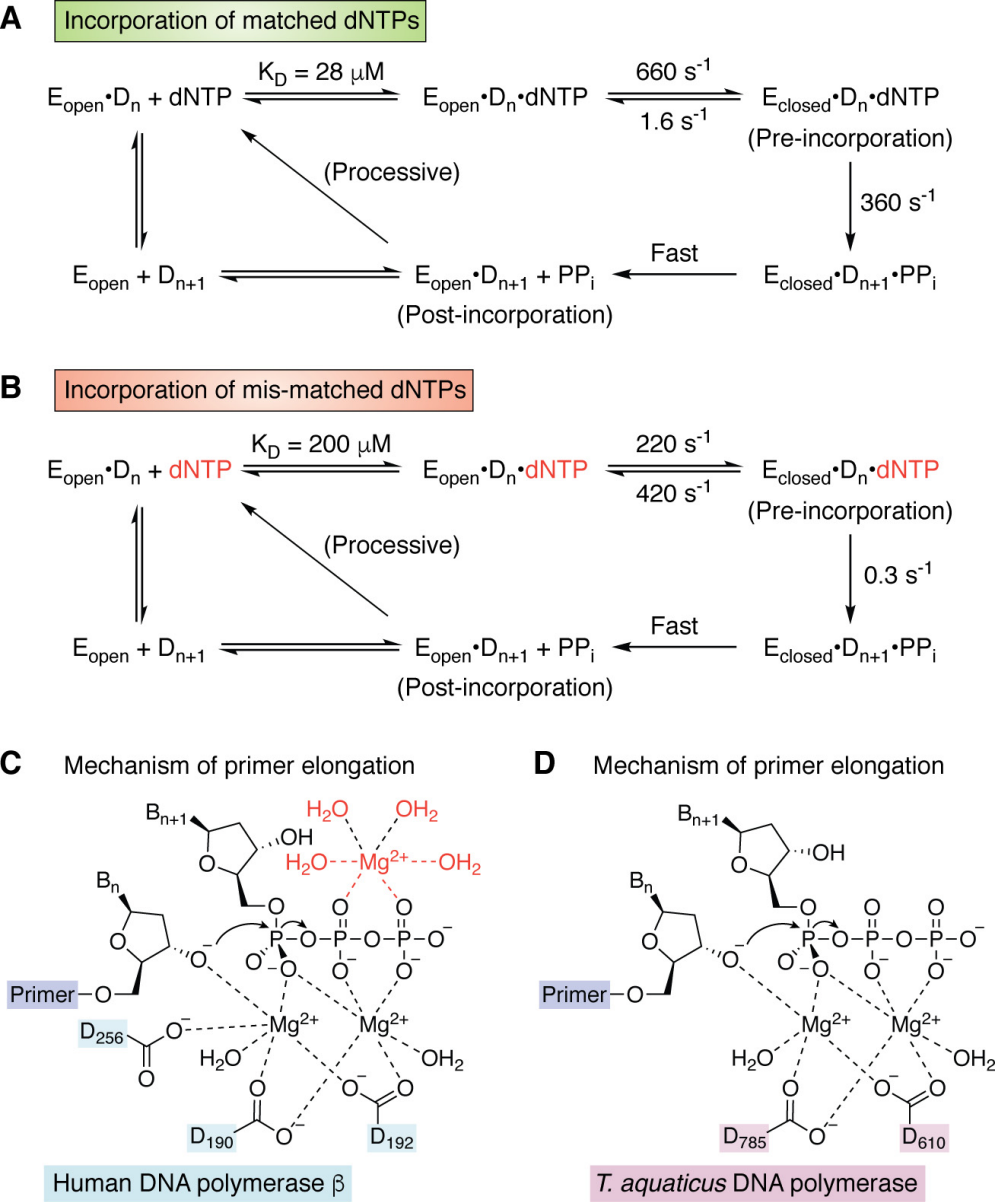
*A*, kinetic scheme for the incorporation of matched dNTPs by T7 DNA polymerase (38, 53). When the incoming dNTP is correct, the enzyme undergoes a rapid conformational change from the open to closed form of the pre-incorporation complex. *B*, kinetic scheme for the incorporation of mis-matched dNTPs by T7 DNA polymerase (38, 53). When the incoming dNTP is incorrect, the rate constant for the chemical step becomes smaller than that for the conformational change back to the open form of the pre-incorporation complex. *C*, chemical mechanism of primer elongation for the 2-Mg (54) and 3-Mg models (55). The location of the third Mg^2+^ ion together with its water ligands is shown in *red*. Residue numbering is for human DNA polymerase β. *D*, chemical mechanism of primer elongation in the active site of *T. aquaticus* DNA polymerase, which lacks one of the carboxylate ligands present in human DNA polymerase β.

Although the coupling reaction catalyzed by DNA polymerases seems superficially straightforward, this simplicity hides many details that remain controversial (30, 34, 50–52). DNA synthesis proceeds in the 5′ to 3′ direction in a primer-dependent reaction requiring Mg^2+^ as a cofactor. In the first step, the enzyme forms an initial binary complex with a DNA duplex composed of a template strand and a shorter, complementary primer strand possessing a 3′-hydroxyl group. dNTPs then bind to the binary complex to give a ternary (or “pre-incorporation”) complex (Fig. 2). Correctly matched dNTPs reside longer in the active site, allowing the enzyme to adopt a “closed” conformation in which the 3′-hydroxyl group in the primer can attack the α-phosphate of the bound dNTP (Fig. 2*A*). This reaction requires the presence of two (53, 56), or possibly three (30, 54, 55), Mg^2+^ ions within the active site of human polymerase β (Fig. 2*C*) or Klentaq (Fig. 2*D*). Subsequent release of PP_i_ generates a binary (or “post-incorporation”) complex in which the primer has been elongated by one nucleobase, which then serves as the substrate for the next templated addition of dNTP. These steps are repeated until no unpaired nucleobases remain in the template strand.

Replicative DNA polymerases, especially those for which DNA binding is the rate-limiting step, exhibit a property referred to as processivity (57). Processivity is a measure of the number of dNTPs incorporated into the growing strand before the template-primer duplex dissociates from the enzyme (58). Processivity, of course, permits these DNA polymerases to achieve high rates of nucleotide incorporation. This process requires translocation of the enzyme along the template-primer or the template-primer along the enzyme (depending on your point of view) during polymerization. We discuss the conformational changes that occur during dNTP incorporation below.

When mismatched dNTPs are present in the ternary complex, kinetic evidence shows that the active site remains in an “open” conformation so that dissociation of the incoming nucleotide can take place prior to formation of the new P–O bond (37, 38) (Fig. 2*B*). Alternatively, should a mismatched dNTP be incorporated, most replicative polymerases include a 3′–5′ exonuclease domain that can remove the mismatch. In this case, the 3′-hydroxyl end of the primer shifts from the polymerase to the exonuclease active site for removal of the mismatched nucleotide (59, 60). This proofreading step is essential for ensuring fidelity during replication by DNA polymerases, which typically make errors every 10^4^ to 10^5^ nucleobases. The 3′–5′ exonucleolytic activity improves this error rate by 2–3 orders of magnitude (61).

**Figure 3.**
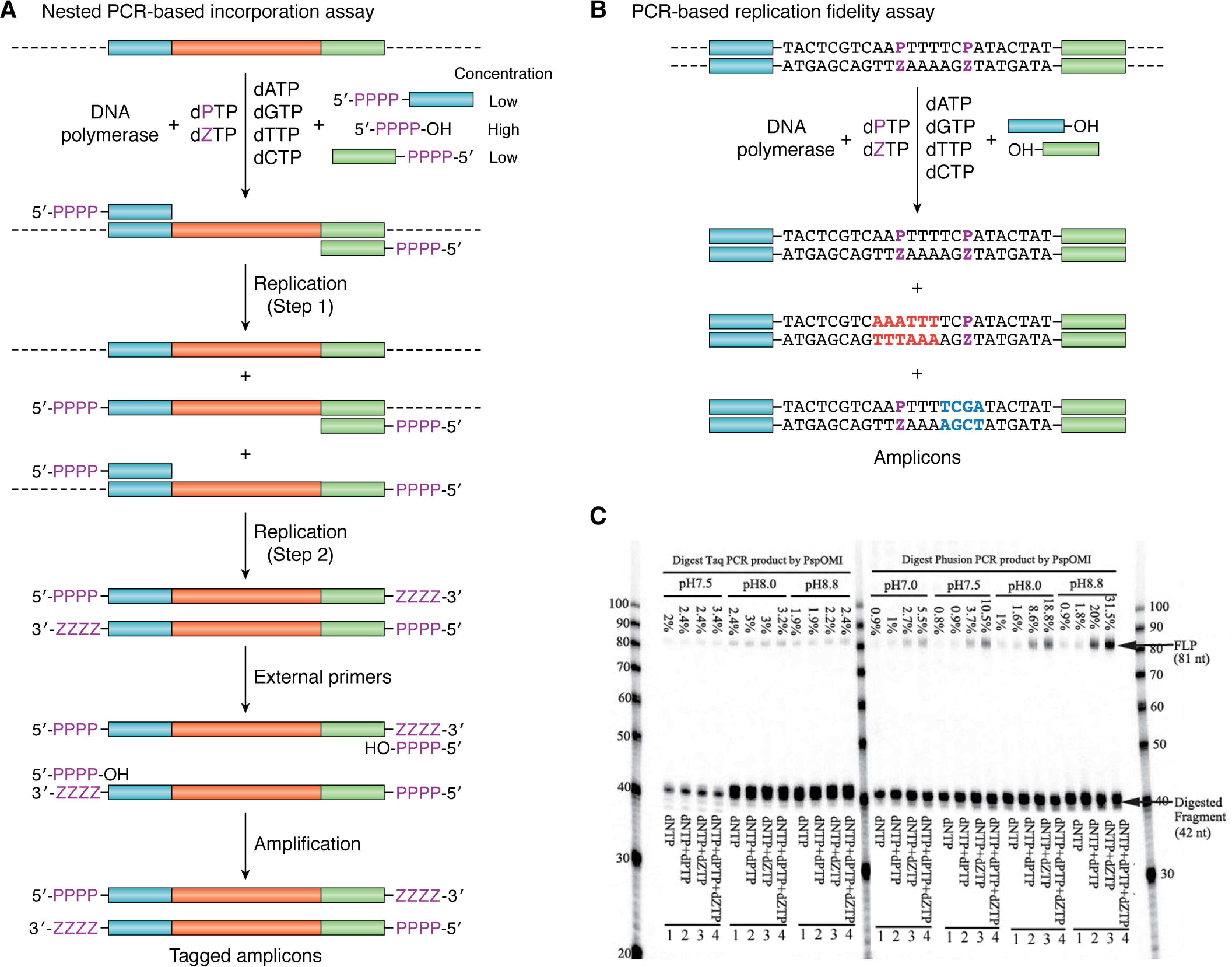
*A*, nested PCR-based incorporation assay for DNA containing Z:P nucleobase pairs using a target DNA sequence (*cyan*-*red*-*green*) composed of only WC base pairs. Replication is initiated by chimeric primers that can bind to the original gene but have UBPs at the 5′-end. These chimeric primers are present at low concentration (*Step 1*). After consumption of the chimeric primers (*Step 2*), amplification is continued by external 5′-PPPP-OH primers (containing only UBPs), which are present at high concentration. As a result, full-length amplicons are produced only if the DNA polymerase can efficiently incorporate UBPs (based on Fig. 2 of Ref. 65). *B*, PCR-based replication fidelity assay for DNA containing **Z**:**P** nucleobase pairs. If the UBP is lost to a T:A base pair, then a Dra restriction site (*red*) is generated in the amplicons. Similarly, if the UBP is lost to a C:G base pair, then an Alu restriction site (*blue*) is produced. *C*, gel showing misincorporation rates in the PCR amplification of template DNA in the presence of dZTP and/or dPTP components using WT Klentaq and WT Phusion DNA polymerases at different pH values (64). Four parallel PCRs were performed followed by digestion with PspOMI for 16 h. The ratio between the amount of full-length product (*FLP*) and all oligonucleotides gives the misincorporation rates shown in the figure. *Lane 1*, negative control PCR amplification of template DNA in the presence of WC dNTPs (200 mm each), followed by digestion with PspOMI. *Lane 2*, five-letter PCR amplification of template DNA in the presence of WC dNTPs (200 mm each) and dZTP (25 mm), followed by digestion with PspOMI. *Lane 3*, five-letter PCR amplification of template DNA in the presence of WC dNTPs (200 mm each) and dPTP (25 mm), followed by digestion with PspOMI. *Lane 4*, six-letter PCR amplification of template DNA in the presence of WC dNTPs (200 mm each), dZTP (25 mm), and dPTP (25 mm), followed by digestion with PspOMI. Taken from Ref. 64 and used with permission.

A variety of assays exist to assess the kinetics of nucleobase incorporation and processivity. These have been extensively reviewed (34, 62), but here we give two examples used in our work to determine either incorporation efficiency (63) or fidelity (64) when DNA polymerases and their variants are challenged with AEGIS UBPs (Fig. 3). For example, incorporation efficiency can be measured by nested PCR using primers tagged at the 5′-end with multiple consecutive UBPs. In this assay, amplicons are formed in the reaction mixture only if the polymerase is capable of replicating the UBPs (Fig. 3*A*).

Assaying fidelity requires the DNA polymerase to replicate a sequence in which UBPs are present (64) (Fig. 3*B*). In the event that the UBP is lost during PCR cycles, two possible unique restriction sites are created in the amplicons, depending on whether the UBP is replaced by T:A or C:G. Incubating the PCR products with two restriction endonucleases therefore gives cleaved products for the amplicons from which the UBP has been lost in both possible transition mutations (Fig. 3*C*). This assay thereby reports on the extent to which polymerase fidelity deviates from 100%. In addition, performing the PCR with different concentrations of the UBP components permits a quantitative assessment of fidelity.

## Obtaining DNA polymerases engineered for altered substrate specificity

Some, albeit limited, success has been reported in modifying the substrate preferences of DNA polymerases by structure-guided mutagenesis studies (66, 67). The complexity of the conformational changes undergone by the polymerase during each catalytic cycle seems to require that variants contain multiple residue replacements. Most successful efforts to obtain DNA polymerases with altered catalytic and/or biophysical properties have therefore selected suitable variants from large libraries (40, 68–72). Examples include several *Taq* polymerase variants evolved for specific functions (Table 1). The fact that several of the amino acid substitutions are found in more than one of these *Taq* variants may ultimately inform rationale design of variants with new functional properties in the future. To date, however, only one of these published variants (ZP Klentaq) has been subjected to structural and dynamic characterizations to determine the impact of each of the various substitutions on the properties of the variant polymerase (see below).

**Table 1:** **Examples of evolved *Taq* DNA polymerase variants** Residues in boldface/italic type are found in two or more of the reported *Taq* DNA polymerase variants. The second column indicates whether variants were generated for specific substrates, such as C2′ modified nucleic acid, or for a specific property, such as thermostability.

*Taq* variant	Substrate/Property	Amino acid substitutions	Reference
SFM4-3	C2′ modification	***I614E/E615G/N583S***/D655N/E681K/***E742Q*/*M747R***	70
AA40	RNA, DNA	***E602V/A608V/I614M/E615G***	73
M1	3′ mismatches	G84A/D144G/K314R/***E520G***/F598L/***A608V/E742G***	74
M4	3′ mismatches	D58G, R74P, A109T, L245R, R343G, G370D, ***E520G, N583S***, E694K, A743P	74
5D4	Hydrophobic bases	V62I, Y78H, T88S, P114Q, P264S, E303V, G389V, E424G, E432G, ***E602G, A608V, I614M***, M761T, M775T	75
H15	Thermostability	K225E/E388V/K540R/D578G/***N583S/M747R***	76
RT-KTQ	m6-A sequencing	L459M/S515R/I638F/***M747K***	69
ZP	Z:P substrates	M444V/P527A/E551E/E832V	65

**Figure 4.**
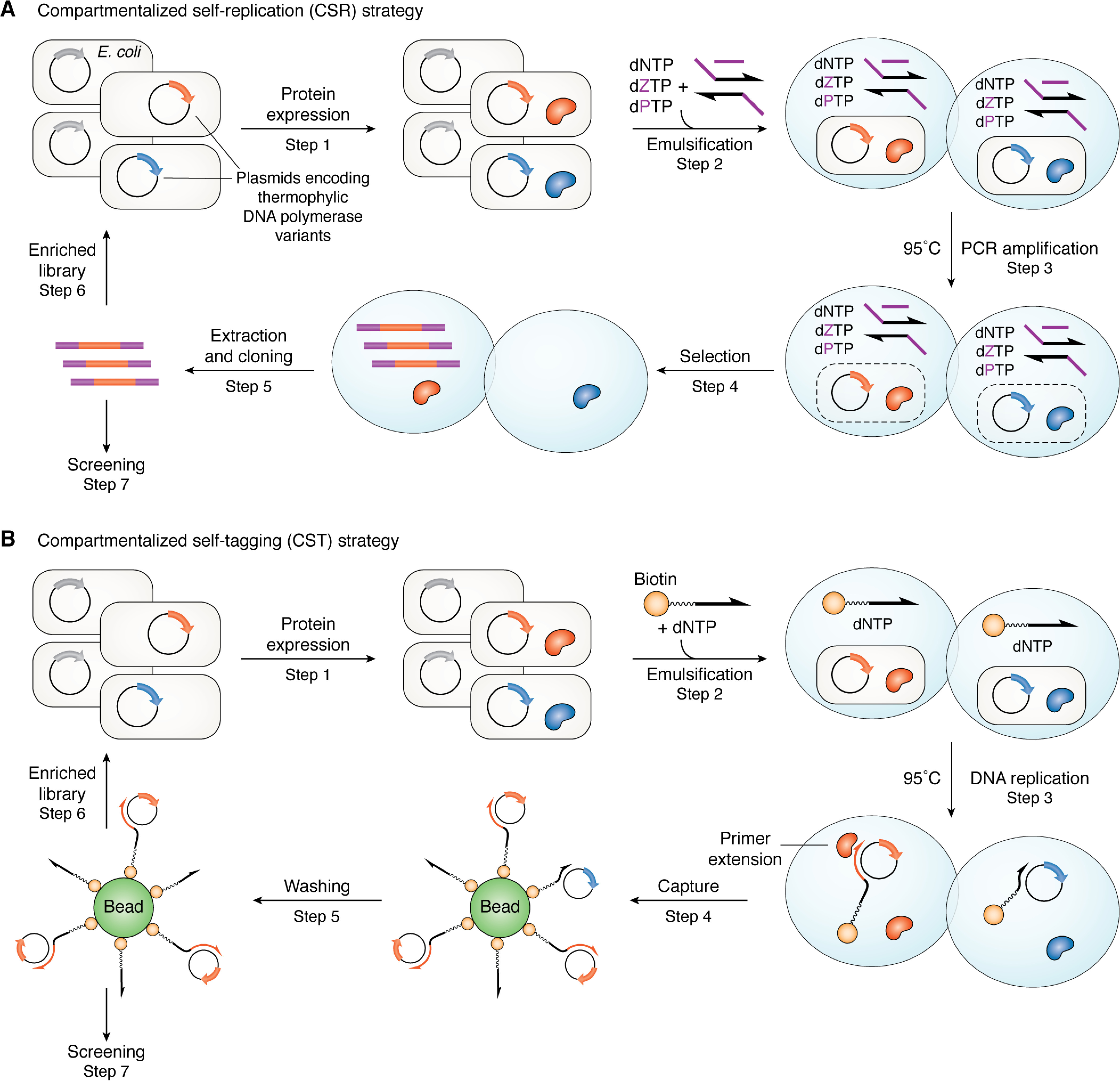
*A*, overview of a modified CSR strategy for identifying DNA polymerase variants capable of replicating AEGIS UBPs from a library. Each *E. coli* cell carries a thermophilic DNA polymerase variant (*Step 1*) and is emulsified with dNTPs and nested primers within a single water droplet. Heating lyses the cells and inactivates endogenous mesophilic polymerases (*Step 2*). PCR amplification is then performed (*Step 3*). Polymerases that can replicate UBPs (*red*) amplify their respective genes, enriching the pool of variant genes in the pool; those that cannot replicate UBPs fail to do so, so their genes are lost (*Step 4*). The amplified genes encoding polymerase variants that can replicate UBPs are extracted after breaking the emulsion and used to create a new library of plasmids (*Step 5*) that can be subjected to a further selection round (*Step 6*). After an appropriate number of selection rounds, the pool of enriched genes can be sequenced (*Step 7*) and used to express polymerase variants that can be characterized using standard *in vitro* methods. Based on Fig. 3 of Ref. 65. *B*, In CST, each *E. coli* cell carries a thermophilic DNA polymerase variant encoded on a plasmid (*Step 1*), which also contains a sequence containing one or more UBPs complementary to a single biotin-linked (“tagged”) primer and is emulsified with dNTPs and the tagged primer within a single water droplet. Heating lyses the cells and inactivates endogenous mesophilic polymerases (*Step 2*). Subsequent DNA replication (*Step 3*) by the thermophilic DNA polymerase variant results in a “biotin-tagged” form of the plasmid containing the gene (*red*) encoding the polymerase variant that can replicate the UBP(s). Polymerases that cannot replicate the UBP (*blue*) will only give weakly stable primer-plasmid complexes. After breaking the emulsion, tagged plasmids are captured onto a bead (*Step 4*) in proportion to the stability of the extended primer-plasmid complex (*Step 5*). The recovered plasmid DNA is then amplified and used in a new round of selection (*Step 6*) or screened (*Step 7*). Based on Fig. S1 of Ref. 78).

Of the plethora of directed evolution strategies, compartmentalized self-replication (CSR) (76, 77) or one of its modifications (40, 68) is often used to obtain engineered DNA polymerases. In CSR, water droplets containing appropriate primers and dNTPs are formed in a water/oil emulsion. Droplets that also contain a single *E. coli* cell carrying a plasmid encoding a single variant of a thermophilic DNA polymerase, such as Klentaq, can then be used to select enzymes with desired properties. In our work (65), we employ the nested PCR strategy described above (Fig. 3*A*) to identify DNA polymerase variants that can replicate UBPs orthogonal to WC nucleobases, such as Z and P, because only these will replicate copies of their gene in the plasmid (Fig. 4). Those variants that cannot incorporate UBPs efficiently will not replicate their genes. Thus, genes encoding DNA polymerases with the desired substrate specificity become represented more in the mixture of DNA amplicons obtained when the emulsion is broken at the end of several rounds of PCR (Fig. 4). The resulting DNA amplicons are inserted into a new set of plasmids that are used to transform *E. coli* for use in a subsequent selection round. After a defined number of rounds, genes encoding DNA polymerase variants with the ability to incorporate UBPs become highly enriched in the library. The set of enriched genes can be sequenced to identify the molecular changes that permit the polymerase variant to replicate the UBP. Standard methods can then be used to express and purify individual variants prior to any detailed evaluation of their fidelity and incorporation efficiencies (63, 64).

The original CSR strategy, however, requires the replication of the entire polymerase gene, which contains more than 2000 nucleobases. Thus, only the most active variants are recovered. Many technical aspects, including the appropriate choice of oil or surfactant containing the water droplets (79, 80), must be optimized if reproducible results are to be obtained (81).

Improved variations of this selection method have therefore been reported by Philip Holliger and co-workers (78, 82), including compartmentalized self-tagging (CST) (Fig. 4*B*). In CST, the positive feedback loop depends on the polymerase “tagging” a plasmid containing its encoding gene by extension of a biotinylated oligonucleotide. As a result, plasmids containing genes that encode DNA polymerases with altered substrate selectivity can be enriched by selective capture onto an affinity column. Clearly, the UBP(s) must be located within a segment of plasmid DNA that is replicated with high efficiency and fidelity by the DNA polymerase variant. In addition, the sensitivity of the assay is higher because CST does not require multiple self-replications of the complete gene encoding the polymerase variant.

## Insights from structural studies of WT and evolved Klentaq DNA polymerase complexes

In contrast to some other critical cellular enzymes and machines, DNA polymerases have been reinvented throughout evolution (32). As a consequence, several distinct structural classes of polymerases provide multiple opportunities to identify an enzyme that will efficiently and faithfully replicate an unnatural base pair. Replicative DNA polymerases have been classified as families A–D, with D being the most recently identified DNA polymerase family from Archaea (32).

**Figure 5.**
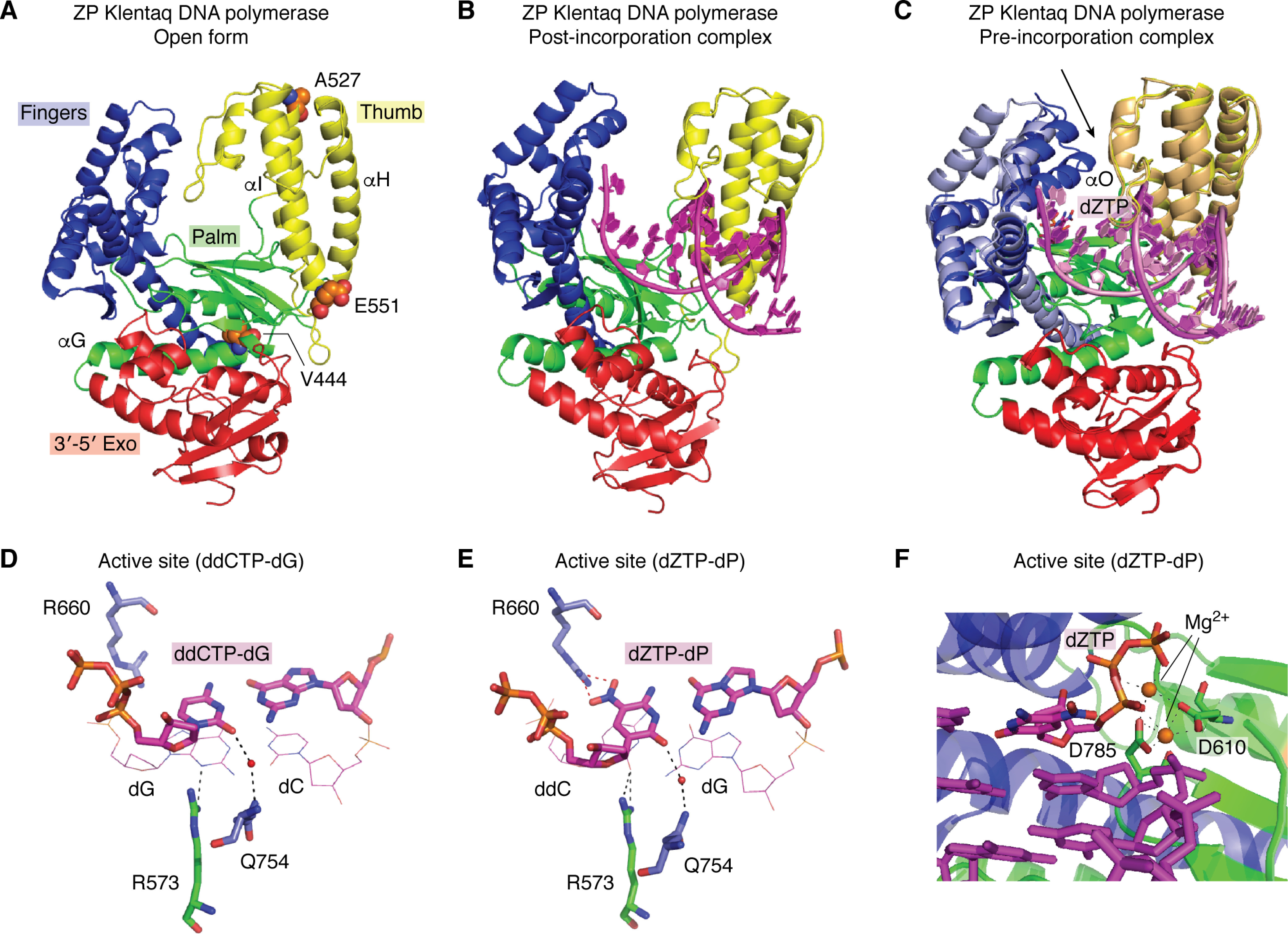
**Structural properties of Klentaq DNA polymerase.** *A*, the open form of ZP Klentaq (from PDB entry 5W6Q), shown in a *cartoon rendering*, includes fingers (*blue*), palm (*green*), thumb (*yellow*), and 3′–5′ exonuclease (*red*) domains. This architecture is conserved in all family A polymerases. Variant amino acid substitutions are shown as *sphere models* (*orange*, carbon; *blue*, nitrogen; *red*, oxygen), M444V, P527A, and D551E. (The fourth amino acid substitution, E832V, is disordered in the structures). α-Helices G, H, and I contribute to dynamic motion in ZP Klentaq. *B*, a similar rendering of ZP Klentaq in the binary, post-incorporation complex (PDB entry 5W6Q) is shown with template-primer, including P:Z in the active site (*magenta cartoon rendering*). *C*, the pre-incorporation complex of ZP Klentaq (PDB entry 5W6K) is shown superimposed on the post-incorporation complex from *B*. The *black arrow* indicates the most significant conformational change associated with the fingers domain (*light blue* for the initial and post-incorporation and *blue* for the pre-incorporation complex). The O helix closes down, forming the active site in the ternary complex with bound dZTP (*magenta stick rendering*). The thumb domains show a slight shift in position (*light orange* for post-incorporation and *yellow* for pre-incorporation complex). The palm and exonuclease domains show no significant differences in overall position in the two complexes. The substrate nucleic acid is shown as a *cartoon rendering* (*pink* in the post-incorporation and *magenta* in the post-incorporation complex). *Close-ups* of hydrogen-bonding interactions in the active site for the WT Klentaq bound to ddCTP-dG and dZTP-dP are shown in *D* and *E*, respectively. In each case, the dNTP is positioned through a water-mediated hydrogen-bonding interaction involving O2 (minor groove edge of the nucleobase) with Gln-754. Similarly, the penultimate primer nucleobase presents either N3 or O2 for hydrogen-bonding to Arg-573 (again a minor groove interaction). In the case of the ZP Klentaq ternary complex (*E*), Arg-660 hydrogen-bonds to the NO_2_ group present on the major groove edge of Z. *F*, the active site including template-primer and dZTP (*magenta stick rendering*), Mg^2+^ ions (*orange spheres*), and coordinating residues Asp-610 and Asp-785 (*green stick rendering*) are shown with a *ribbon rendering* of the enzyme, palm in *green* and fingers in *blue*.

To date, naturally occurring family A and B DNA polymerases have been used to successfully replicate single UBPs. Family A polymerases are found primarily in bacteria; their peptide fold is described as including fingers, palm, and thumb domains associated with polymerase activity (Fig. 5*A*). They also have 3′–5′ exonuclease and 5′–3′ exonuclease domains, the second removing the RNA primers required for lagging strand synthesis (32). In Klentaq, the *Thermus aquaticus* DNA polymerase (83), corresponding to the Klenow or large fragment of the enzyme (84), the N-terminal 5′–3′ exonuclease domain is absent, and the 3′–5′ exonuclease domain lacks activity. In contrast, family B DNA polymerases, found in all archaea, include a 3′–5′ exonuclease domain and a polymerase domain, which is related to that in family A polymerases (32).

Hydrophobic nonhydrogen-bonding pairs developed by Romesberg (NAM:TPT3) (8, 9) and Hirao (Ds:Px) (10, 11) have been replicated with selectivities exceeding 99% using DeepVent DNA polymerase (family B) (85) or a combination of DeepVent DNA polymerase and *Taq* DNA polymerase (family A) (14), respectively. It has proven much more difficult, however, to replicate templates containing consecutive hydrophobic nucleobases. The problem appears to be caused, in part, by disruptions to the DNA double helix resulting from the presence of hydrophobic UBPs (86). For example, the dNaM:d5SICS pair adopts an intercalative stacking mode in duplex DNA, as seen in both NMR structures and binary template-primer Klentaq complexes (87, 88). At least 6 natural base pairs must separate Ds-Px pairs if replication is to be successful (89); as yet there is no report of DNA containing consecutive NaM:PTP3 (or structurally related hydrophobic UBPs) being replicated. Of course, the necessity of replicating more than one UBP, either consecutive or separted by standard WC nucleobases depends on the biotechnological application (15).

A truly artificial genome would presumably include consecutive UBPs just as natural genomes include runs of sequential A:T or G:C pairs; cells possessing such a genome would require a DNA polymerase that can replicate through these regions of sequence. We have shown that up to 4 consecutive Z:P pairs can be incorporated by WT Klentaq polymerase (63); these UBP tetrads do not adversely impact the ability of duplex DNA to adopt A- and B-conformations (90, 91). Unfortunately, the efficiency of Z:P incorporation by WT Klentaq is only 99.8% per theoretical PCR cycle (63), meaning that the UBP is easily lost during PCR-based applications. An engineered Klentaq variant (ZP Klentaq), exhibiting improved fidelity and Z:P incorporation efficiency was, however, obtained using CSR (Fig. 4*A*) (65). Remarkably, ZP Klentaq contains only four amino acid substitutions, M444V, P527A, D551E, and E832V, all of which are located distal from the active site (Fig. 5*A*).

In family A DNA polymerases, a large conformational change in the fingers domain occurs upon binding of the complementary dNTP, resulting in the formation of a closed complex that facilitates incorporation (Fig. 5). Of the structurally characterized DNA polymerases related to Klentaq at the sequence level (Table 2), only three have been captured in both pre- and post-incorporation complexes: ZP Klentaq (92), WT Klentaq (6), and WT *Geobacillus kaustophilus* DNA polymerase (93). Incorporation of nucleotides by the *Geobacillus* enzyme exhibits a fingers domain closure angle of 37°. In contrast to the *Geobacillus* enzyme, incorporation of natural dNTPs by WT Klentaq involves a much larger conformational change in which the fingers domain rotates by ∼59° (Fig. 5, *B* and *C*). In the closed conformation of the polymerase, correctly paired nucleotides are selected by hydrogen bonding and size complementarity. Despite retaining these features, Z:P pairs are more efficiently and faithfully incorporated by ZP Klentaq, in which the fingers close down by ∼64° (Table 3). Protein-nucleic acid interactions in both pre- and post-incorporations for ZP Klentaq are similar to those observed in the analogous WT complexes, suggesting that the evolved polymerase closely mimics the WT enzyme in this regard (Fig. 5, *D* and *E*).

**Table 2:** **Structure-based alignments of *Taq*-related DNA polymerases** Binary and apo structures were superimposed with ZP Klentaq binary complex (5W6Q:CEF, chain identifiers); ternary complexes were superimposed with ZP Klentaq ternary complex (5W6K). RMSD, root mean square deviation

PDB	Type	Source	No. of aligned Cα atoms	RMSD	Sequence identity
				*Å*	%
5DKU	Apo	*Plasmodium*	418/521	2.8	24.6
4DQS	Binary	*G. kaustophilus*	486/521	1.8	42.8
1KFD	Apo	*E. coli*	429/521	1.9	42.7
2BPD	Binary	*Geobacilllus stearothermophilus*	488/521	1.8	43.4
4XVI	Binary	*Homo sapiens* (Pol ν)	439/521	2.5	31.9
6VDE	Apo	*Mycobacterium smegmatis*	487/521	2.1	40.0
4 × 0Q	Ternary	*H. sapiens* (Pol θ)	443/522	2.4	29.6

**Table 3:** **Comparisons of binary and ternary complexes** ZP tern, the ternary complex of ZP Klentaq; ZP Bin C, the binary complex of ZP Klentaq. *, Klenow fragment of *G. kaustophilus* DNA polymerase I. All other structures correspond to the Klentaq, the Klenow fragment of *Thermus aquaticus* DNA polymerase.

PDB file 1	PDB file 2	Fixed domain	Moving domain	Rotation angle	Translation	Closure	Bending residues
				*degrees*	*Å*	%	
5W6K ZP tern	5W6Q ZP Bin C	299–637	638–671	63.6	−1.7	88.8	632–638
		672–829					670–704
3RTV	3SZ2	295–637	638–671	59.6	−1.5	79.4	626-638
		672–830					669–696
3KTQ	4KTQ	296–636	637–670	58.2	−1.9	67.8	626–637
		671–829					670–686
4DQQ*	4DQS*	300–679	680–716	36.6	−0.7	86.6	676–680
		717–874					712–744

The post-incorporation ZP Klentaq complex (PDB entry 5W6Q) (92) also exhibits increased thumb domain motion (∼4°) relative to the analogous WT Klentaq complex (PDB entry 3SZ2) (6). Although Z:P pairs are readily accommodated in both B- and A-form DNA duplexes (90, 91) and retain standard features (groove widths, base stacking parameters, etc.) consistent with these helical forms, modeling of a post-incorporation complex of P:Z bound to the WT Klentaq suggested that some structural alterations within the active site would be required to accommodate the UBP (92). The two base pairs closest to the site of incorporation of the next dNTP exhibit A-form, which is significantly wider than B-form. This feature of the active site may allow WT Klentaq to accommodate a single hydrophobic UBP, which is wider than hydrogen-bonded pairs (6, 94).

Of the amino acid substitutions in ZP Klentaq, M444V was considered to be the best candidate for providing access to increased relative domain motion as compared with the WT enzyme. Residue 444 resides within the hydrophobic core of the palm domain, which serves as the command center for the enzyme. Both the fingers and thumb domains are connected to the palm domain. In addition, the essential catalytic residues Asp-610 and Asp-785, which coordinate Mg^2+^ and the incoming dNTP in the active site of the enzyme, reside in the palm domain (Fig. 5*F*). Substitution of a Val for Met at this position creates open space within the core that could potentially translate into increased motion of the fingers and thumb domains.

## Augmenting crystallography with molecular dynamics simulations of natural and evolved DNA polymerases

**Figure 6.**
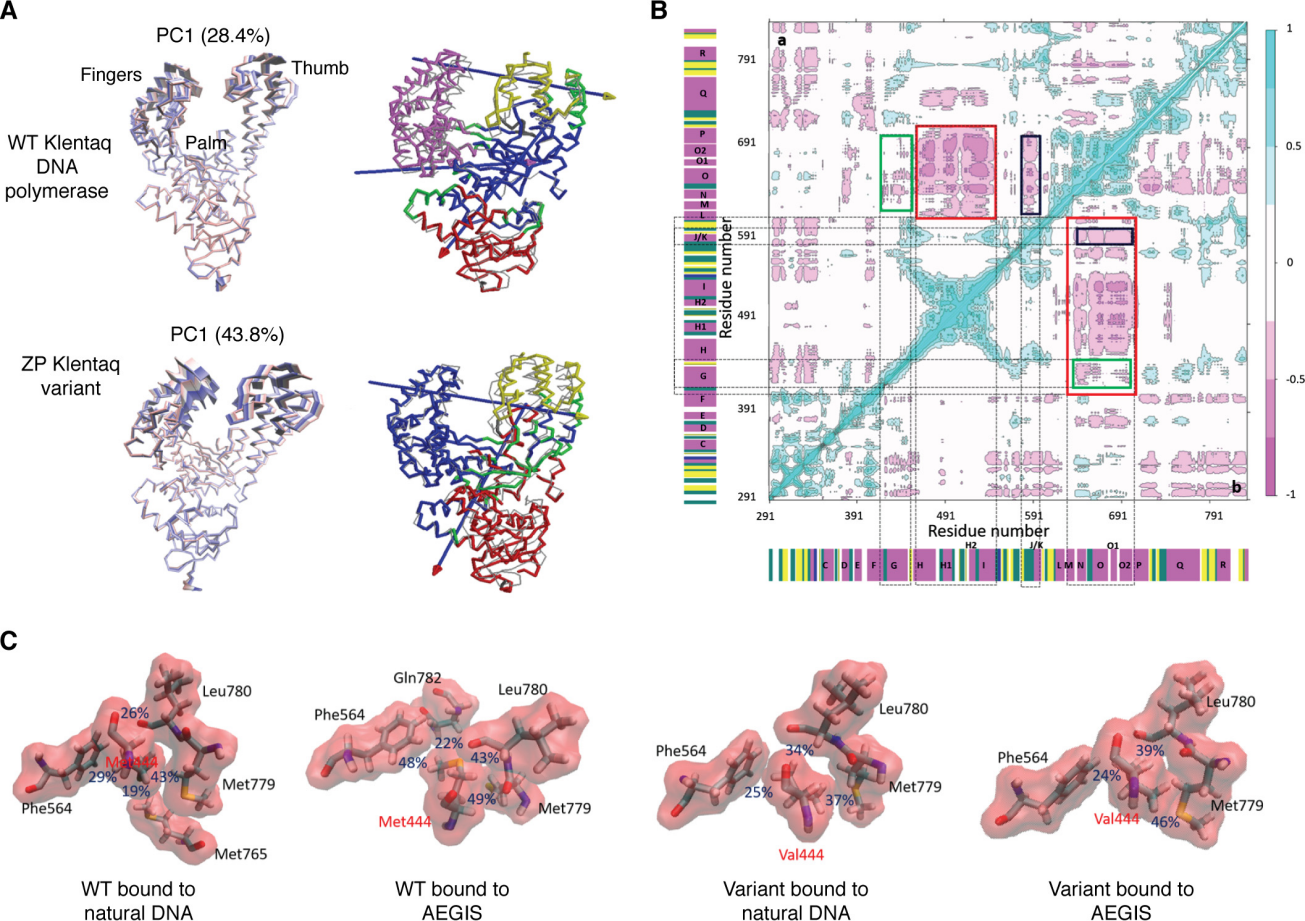
*A*, two complementary *cartoon representations* of motions in the binary complexes of WT Klentaq (*top*) and ZP Klentaq variant (*bottom*). *Left*, the major principal component (PC1) (99) at early (*red*), intermediate (*white*), and late (*blue*) stages of the sampled MD trajectory, showing that the dynamics of the fingers and palm domains are altered when WC and AEGIS template-primer strands are bound for the two DNA polymerases. *Right*, domain motions of the polymerase complexed to AEGIS DNA computed using DynDom (100). For reference, the WT Klentaq/WC DNA binary complex (PDB entry 3SZ2) is shown in *gray*. Each axis of domain motion is indicated by an *arrow*, the *head* of which is *colored* to represent the moving domain (*red*, *yellow*, or *purple*). The fixed domain (*blue*) and regions that bend during the motion are shown in *green*. *B*, dynamic cross-correlation maps computed with bio3d (101) for the AEGIS DNA-containing binary complex of WT Klentaq (*top triangle*) and ZP Klentaq (*bottom triangle*). Correlated (range: 0.25–1) and anti-correlated (range: −0.25 to −1) motions are *colored* from *light* to *dark blue* and *pink*, respectively. Areas rendered in *white* correspond to noncorrelated motions (range: −0.25 to 0.25). Secondary structural elements are also included on the map. *Colored boxes* show interconnected residue networks with altered motions in the two polymerases. *C*, altered interactions between residue 444 and surrounding side chains in the hydrophobic core observed in the MD trajectories of the WT Klentaq/Watson–Crick DNA binary complex (*left*), WT Klentaq/AEGIS DNA binary complex (*middle left*), ZP Klentaq/Watson–Crick DNA binary complex (*middle right*), and ZP Klentaq/AEGIS DNA binary complex (*right*). The percentages of occurrence of specific interactions in the MD trajectory are also shown. Based on Fig. 7 of Ref. 98).

Molecular dynamics (MD) simulations are a well-validated method of studying protein dynamics (95) and have yielded important information about conformational changes undergone by DNA polymerases (96, 97). Our recent work also demonstrates that MD simulations can be used to understand how substitutions far from the active site might alter substrate selectivity (98). For example, microsecond trajectories have shown how the dynamic motions of ZP Klentaq differ from those of WT Klentaq in the binary complex containing template-primer DNA duplexes (Fig. 6*A*). They can also highlight correlated motions in networks of residues that are critical to altered domain motions in the variant DNA polymerase (Fig. 6*B*) (98), which permit the engineered enzyme to bind WC and Z:P-containing template-primer duplexes in an equivalent fashion, thereby increasing the efficiency of UBP incorporation. This seems to be a consequence of replacing Met-444 and Asp-551 at the base of the thumb domain by valine and glutamate, respectively, which allows the fingers, palm, and thumb domains in ZP Klentaq to move into position about the AEGIS template-primer duplex. Thus, replacing Met-444 by valine in the hydrophobic core changes the populated interactions with adjacent residues (Phe-564, Met-779, and Leu-780) in the hydrophobic core (Fig. 6*C*) with the consequence that the G helix becomes more flexible in the variant compared with the WT polymerase (98).

As well as modeling how amino acid substitutions might impact long time-scale motions, such as domain reorientations, MD simulations can illuminate specific molecular changes that have the greatest impact on the altered catalytic biophysical and catalytic properties of the DNA polymerase variant. In the case of ZP Klentaq, the role of each of the substitutions (M444V, P527A, D551E, or E832V) was determined using MD simulations of the four single-point Klentaq variants (98). These calculations showed that replacing Met-444 in the hydrophobic core of the palm domain with valine makes a significant contribution to altering dynamic motions in ZP Klentaq. In addition, changing Asp-551 to glutamate alters the interactions of the H and I helices at the base of the thumb domain, which affects G helix flexibility (Fig. 6*B*) (98).

Care must be taken to perform MD simulations on the correct form of the enzyme. For example, introducing valine in place of Glu-832, which breaks a salt bridge with Arg-596, has little impact on the dynamics of the ZP Klentaq/DNA binary complex. However, it does alter protein/DNA interactions. This substitution may therefore exert its effects in the pre-incorporation (ternary) complex during dNTP binding and incorporation.

MD simulations have also provided interesting insights into the “closing mechanism” of DNA polymerase I prior to the incorporation of WC nucleobases (102) and the temperature-dependent activity of a Klentaq variant in which Ile-707, located 20 Å away from the active site, is replaced by leucine (103). This I707L Klentaq variant exhibits low activity at 37 °C but efficiently replicates DNA at 68 °C, permitting improved PCR amplification of difficult sequences. MD simulations show that the I707L Klentaq variant is almost immobile at 37 °C but exhibits increased flexibility at 68 °C. In contrast, the WT enzyme exhibits less pronounced dynamic changes in simulations performed at 37 and 68 °C. Thus, it is likely that the reduced activity of the I707L Klentaq variant is associated with the enzyme remaining in its closed conformation at lower temperatures (*i.e.* dNTP binding is blocked) and that the pre-incorporation complex cannot form. As observed for ZP Klentaq (98), the molecular origins of this behavior could be traced to changes in the hydrophobic core; replacing isoleucine by leucine results in an alternate conformation of an adjacent phenylalanine (Phe-749), thereby repositioning the O and O1 helices. As a consequence, the active site becomes filled with nucleobases in the template overhang, thereby blocking entry of the incoming dNTP.

## Outlook

Realizing the full potential of UBPs in the creation of new research, diagnostic, and therapeutic tools will ultimately depend on our ability to identify DNA polymerase variants that can faithfully and efficiently replicate those UBPs. Many of these applications will necessarily require enzymes that are active at the high temperatures used in PCR, limiting the pool of candidate DNA polymerases primarily to family A and family B members. For other applications, such as using dNaM-dTPT3 (Fig. 1) in semi-synthetic organisms that introduce noncanonical amino acids into specific proteins or the isothermal amplification of AEGIS UBPs (104), DNA polymerases that function at 37 °C, such as those in *E. coli*, will be adequate as starting points for structure-based engineering or library construction and selection.

To date, progress in engineering DNA polymerases has been encouraging (105). In our work aimed at improving the ability of these enzyme to replicate UBPs (65, 92, 98), ZP Klentaq takes advantage of increased domain flexibility to improve the replication efficiency of Z:P pairs, even though both WT Klentaq and ZP Klentaq grip the template-primer duplex in a similar manner prior to and following incorporation of the AEGIS UBP.

Combining X-ray crystallography with MD simulations yields two important conclusions. First, not all substituted residues impact the properties of the evolved enzyme equally. Second, substitution of a key residue, such as Met-444, can result in increased relative domain motion, even in an enzyme that exhibits a very large conformational change during catalysis. It is this altered motion that allows ZP Klentaq to incorporate UBPs more readily than the WT enzyme.

**Figure 7.**
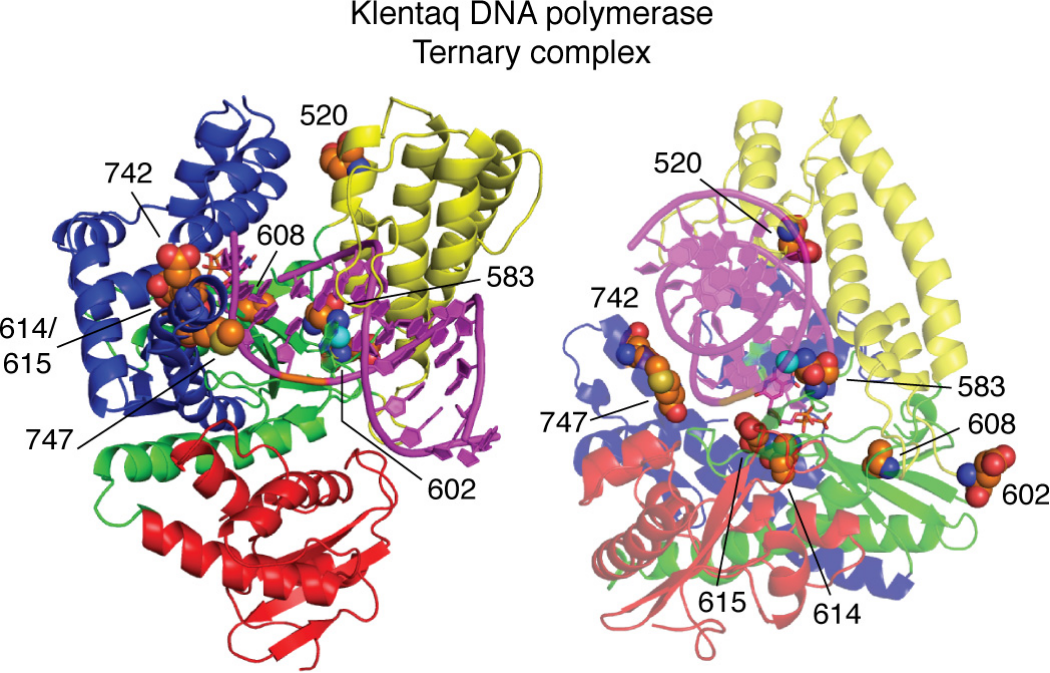
**A *cartoon rendering* showing two views of the ternary complex of Klentaq (fingers, *blue*; palm, *green*; thumb, *yellow*; exonuclease domain, *red*) with amino acid substitutions common to evolved Klentaq variants shown in van der Waals *sphere renderings* (carbon, *orange*; oxygen, *red*; nitrogen, *blue*; sulfur, *yellow*).** None of the side chains of these residues are involved in direct hydrogen-bonding interactions with the template-primer or dNTP in the active site. Most of these residues are distant from substrate template-primer and dNTP, although the main-chain NH of Glu-615 hydrogen-bonds to the 3′-OH of the dNTP deoxyribose ring, and Asn-583 makes a water-mediated hydrogen bond to the backbone of the template strand. In addition, Ile-614 and Glu-615 are within van der Waals contact distance of the dNTP bound to the active site, and Met-747 is within contact distance of the template strand.

CSR, CST, and related strategies for the directed evolution of DNA polymerases, which typically produce variants containing several amino acid substitutions, might therefore be improved by targeting fewer residues outside the active site for variation. Coupling structure determinations, MD simulations, and enzymatic characterization will provide guiding principles for CSR library design and facilitate rational approaches to optimize family A DNA polymerases to replicate multiple UBPs. Such work can be guided by understanding amino acid substitutions already identified in Klentaq variants (Table 1); for example, Asn-583, Ile-614, and Met-747 are substituted in three of eight variant enzymes, whereas Glu-520, Glu-602, Ala-609, Glu-615, and Glu-742 are substituted in two of eight variants. None of these residues are involved in direct hydrogen-bonding interactions with the substrate template-primer or the dNTP in the active site (Fig. 7). The past evolutionary history of DNA polymerases may also prove useful for selecting specific sites for variation (42, 106).

We also note that whereas most engineering efforts to date have focused exclusively on improving the ability of DNA polymerases to incorporate UBPs with little consideration for exonuclease activity, a *Thermococcus gorgonarius* DNA polymerase variant selected to replicate xeno-nucleic acids contains two amino acid substitutions that inactivate the 3′–5′ exonuclease site (107). In theory, incorporation of UBPs that slow down polymerization would allow the substrate to be positioned within the exonuclease active site for proofreading. Whether the exonuclease would efficiently process the newly incorporated unnatural nucleobase or not has yet to be fully investigated. It is possible that engineered DNA polymerases may have to possess optimized exonuclease function as well as improved efficiencies for UBP incorporation to maintain artificial genomes.

In summary, the next generation of useful DNA polymerases must (i) replicate more than one UBP with rates and fidelities similar to those observed for WT DNA polymerases when replicating WC dNTPs and (ii) exhibit no significant pausing following UBP incorporation to avoid invoking proofreading mechanisms. These properties will permit the fidelity of these engineered enzymes to approach 99.99%, allowing them to generate full-length replication products in applications involving multiple rounds of PCR. We suggest a strategy for obtaining these DNA polymerase variants that builds on studies of ZP Klentaq, in which a combination of X-ray crystallography and computer simulations showed the importance of a few key residues for conferring increased dynamic motion in the thumb and fingers domains, thereby improving UBP incorporation efficiency. In this approach, altering the properties of an evolved DNA polymerase variant by introducing additional amino acid substitutions will take advantage of a fundamental, structure-based understanding of how dynamic changes in residue “networks” impact kinetic properties (108, 109).
